# Can traditional birth attendants be trained to accurately identify septic infants, initiate antibiotics, and refer in a rural African setting?

**DOI:** 10.9745/GHSP-D-14-00045

**Published:** 2014-08-31

**Authors:** Christopher John Gill, William B MacLeod, Grace Phiri-Mazala, Nicholas G Guerina, Mark Mirochnick, Anna B Knapp, Davidson H Hamer

**Affiliations:** a Boston University School of Public Health, Boston, MA, USA; b Zambia Anglican Council, Lusaka, Zambia; c Women and Neonates Hospital, Providence, Rhode Island, USA; d Boston University School of Medicine, Boston, MA, USA

## Abstract

**Background:**

Neonatal sepsis is a major cause of neonatal mortality. In populations with limited access to health care, early identification of bacterial infections and initiation of antibiotics by community health workers (CHWs) could be lifesaving. It is unknown whether this strategy would be feasible using traditional birth attendants (TBAs), a cadre of CHWs who typically have limited training and educational backgrounds.

**Methods:**

We analyzed data from the intervention arm of a cluster-randomized trial involving TBAs in Lufwanyama District, Zambia, from June 2006 to November 2008. TBAs followed neonates for signs of potential infection through 28 days of life. If any of 16 criteria were met, TBAs administered oral amoxicillin and facilitated referral to a rural health center.

**Results:**

Our analysis included 1,889 neonates with final vital status by day 28. TBAs conducted a median of 2 (interquartile range 2–6) home visits (51.4% in week 1 and 48.2% in weeks 2–4) and referred 208 neonates (11%) for suspected sepsis. Of referred neonates, 176/208 (84.6%) completed their referral. Among neonates given amoxicillin, 171/183 (93.4%) were referred; among referred neonates, 171/208 (82.2%) received amoxicillin. Referral and/or initiation of antibiotics were strongly associated with neonatal death (for referral, relative risk [RR] = 7.93, 95% confidence interval [CI] = 4.4–14.3; for amoxicillin administration, RR = 4.7, 95% CI = 2.4–8.7). Neonates clinically judged to be “extremely sick” by the referring TBA were at greatest risk of death (RR = 8.61, 95% CI = 4.0–18.5).

**Conclusion:**

The strategy of administering a first dose of antibiotics and referring based solely on the clinical evaluation of a TBA is feasible and could be effective in reducing neonatal mortality in remote rural settings.

## Abstract (precis)

Despite having limited training, these TBAs were able to accurately identify critically ill neonates, initiate treatment in the field, and refer for further care. Given their proximity to the mother/infant pair, and their role in rural communities, training and equipping TBAs in this role could be effective in reducing neonatal mortality.

## INTRODUCTION

Neonatal mortality accounts for about 40% of all childhood mortality in low- and middle-income countries. Neonatal sepsis, birth asphyxia, and neonatal hypothermia are responsible for the largest number of preventable deaths.^1^^–^^3^ These conditions are particularly difficult to manage in remote rural settings where home deliveries predominate. In such settings, traditional birth attendants (TBAs) provide 20%–40% of obstetrical care.^4^ Given their close access to the mother/infant pair, strengthening TBAs' capacity could be effective in reducing neonatal mortality.

The Lufwanyama Neonatal Survival Project (LUNESP) was a community-based effectiveness trial in Zambia that assessed the impact of a package of TBA-delivered interventions on all-cause mortality through postpartum day 28.^5^^,^^6^ In LUNESP, TBAs were randomized either to continue their existing standard of care (controls) or to receive training and supplies enabling interventions targeting key preventable causes of neonatal mortality (intervention): birth asphyxia, hypothermia, and neonatal sepsis.^7^ The intervention had 2 components: (1) a simplified version of the neonatal resuscitation protocol (NRP), which targeted deaths from birth asphyxia and neonatal hypothermia; and (2) administration of oral antibiotics with facilitated referral (AFR) to a rural health center, which aimed to reduce deaths from neonatal sepsis.

The LUNESP interventions reduced all-cause day-28 mortality among live-born neonates by nearly half (relative risk [RR] = 0.45, 95% confidence interval [CI] = 0.33–0.9), with the largest reductions during the first 48 hours of life (7.8 deaths/1,000 live births vs. 19.9 deaths/1,000 live births, RR = 0.4, 95% CI = 0.19–0.83).^6^ However, mortality also trended lower during weeks 2 to 4 (RR = 0.47, 95% CI = 0.20–1.11). A late effect of NRP is unlikely to account for this but is consistent with a benefit from AFR. To explore this further, we conducted a secondary analysis focusing on the process by which the intervention TBAs identified, treated, and referred neonates to receive the AFR intervention. We addressed the following issues:

1. How often did the intervention group TBAs refer, and how often was referral coupled with the first dose of amoxicillin (or vice versa)?
2. What were the clinical indications cited for referrals, and how accurately did these predict a fatal outcome for the infant?
3. Is there evidence that TBAs were exercising reasonable clinical judgment in the implementation of the AFR intervention?

Interventions reduced all-cause day-28 mortality among live-born neonates by nearly half.

## METHODS

LUNESP was a cluster-randomized controlled clinical trial conducted between June 2006 and November 2008 in Lufwanyama District, a vast, sparsely populated, rural, and poorly developed region in Zambia's Copperbelt Province. Full details of the LUNESP study have been published previously.^6^^,^^7^ LUNESP was registered on ClinicalTrials.Gov as NCT00518856, with ethical oversight by Boston University Medical Center and the Tropical Diseases Research Centre in Ndola, Zambia. At the time of the LUNESP study, the district included 12 health posts staffed by midwives or clinic officers; there were no physicians and no hospitals. Initially, 120 Zambian TBAs who had previously undergone basic obstetrical training (see below) were randomized 1:1 to intervention or control arms. Control TBAs were trained on reporting aspects of the study, but otherwise they continued their existing care; intervention TBAs received training in NRP and AFR. For this analysis, we used data pertaining to the 60 intervention TBAs.

Detailed information about the TBAs' baseline obstetrical training and the additional training provided for LUNESP has been published in a separate methods paper.^7^ In brief, prior to LUNESP, TBAs already working in Lufwanyama were recruited and registered by the Lufwanyama District Health Management Team. These TBAs were women who had already practiced as TBAs informally for many years but had been nominated by their local village health committees to undergo standardized training on the basis of their perceived skills and value by their community. With that said, this was not synonymous with higher levels of education. Among the TBAs in this analysis, only 17% had received any secondary education, and many could not read or write. All such TBAs received basic obstetrical training, focusing primarily on clean delivery practices, the use of a clean delivery kit with every delivery, and indications for referral (for example, high-risk pregnancies and danger signs emerging during labor). In most cases, this training was provided by the Lufwanyama DHMT itself, but many TBAs were trained instead or in addition by local nongovernmental organizations working in Lufwanyama, and such trainings varied in their duration and intensity. As such, these TBAs served as auxiliary community health workers (CHWs), supported and registered by the local DHMT, and thus could be described properly as “trained TBAs.”

The LUNESP trainings, focusing on AFR and the neonatal resuscitation interventions, were highly standardized. These began with 2 week-long workshops, followed by refresher workshops lasting 2–3 days each, every 3–4 months for the duration of the study. At each workshop, TBAs received group instruction about the interventions, which, due to low levels of literacy, was all done verbally without any supporting text materials. After these combined didactic sessions, TBAs were sorted into working groups of 5–6 for skills training. Each of the TBAs had to complete all steps of the interventions perfectly for the group to graduate. Once all groups had passed, the TBAs underwent one-on-one observed standardized clinical examinations with the master trainer. Only after 100% of the TBAs had passed this final step was the workshop concluded. Because the TBAs' activities (deliveries and AFR interventions) occurred across the vast expanse of Lufwanyama district, there was no opportunity for a supervisor to physically attend these events. Thus, the competence of the TBAs to perform the study interventions was based on their performance during the workshops.

For the AFR intervention, TBAs were trained to identify signs/symptoms of potential severe neonatal infection and neonatal sepsis. These criteria are commonly observed in septic neonates, or those with focal infections at risk for developing sepsis, and were based predominantly on the World Health Organization's (WHO's) Young Infant Clinical Signs Study.^8^ Specific categories of signs/symptoms were:

- Generalized/behavioral changes (lethargy, irritability, poor feeding, sleepy or difficult to arouse, hypotonia, dehydration)
- Temperature instability (too hot or cold)
- Respiratory distress (tachypnea, chest indrawing or retractions, cough, any “breathing difficulty”)
- Central nervous system (CNS)-specific (seizure, bulging fontanel)
- Gastrointestinal (GI)-related (vomiting, diarrhea, abdominal distension)
- Focal infection (skin or umbilical erythema or pustules)

**Figure:**
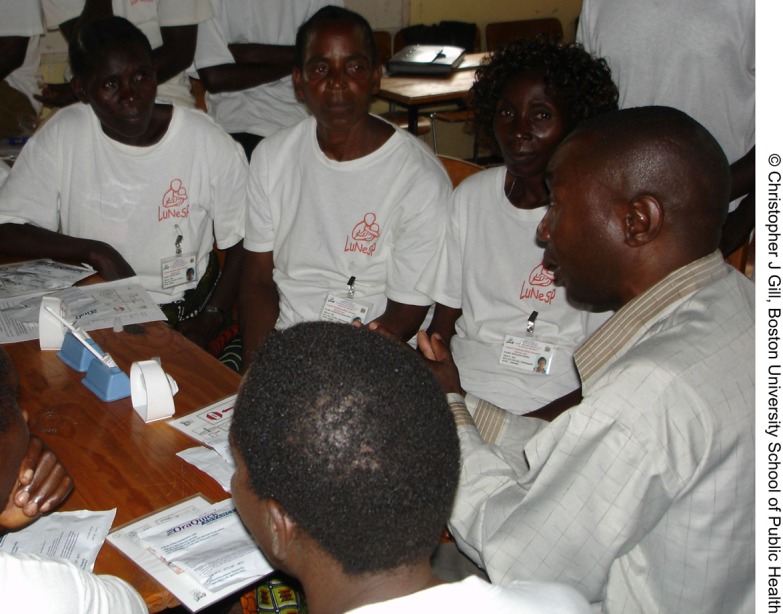
TBAs from Lufwanyama District, Zambia, attend a LUNESP training workshop to improve neonatal survival.

From these, a set of 14 specific criteria were reported by TBAs. In addition, the TBA could trigger the intervention if either the mother or the TBA felt that the baby “appeared ill,” even if no other criteria were met; this brought the total number of criteria to 16.

If any criteria were met, the TBAs were to prepare a slurry of 500 mg amoxicillin using chlorinated water, to administer as much as the infant would accept, and to then encourage the mother to immediately take the infant to the nearest rural health center (RHC), ideally accompanying the mother/infant pair. Amoxicillin use was always to be coupled with referral. A separate team of data collectors interviewed the mothers at the neonates' first and fourth weeks of life documenting TBA referrals and/or amoxicillin use, reasons for referrals, final infant vital status, and the timing of any deaths.

The analysis set comprised all live-born neonates delivered by intervention TBAs with valid data through at least the week-1 visit. We generated descriptive statistics identifying the frequency of TBA referrals, the reasons for AFR, the concordance between RHC referral and amoxicillin administration (and vice versa), the survival rates of referred neonates, and the frequency with which referred mother/infant pairs completed the referral. A “completed referral” was defined as a referred infant who was taken to an RHC for clinical evaluation.

Where appropriate, we conducted bivariate analyses to generate relative risks and 95% confidence intervals for death. Due to the relatively small number of deaths (n = 43), we did not conduct multivariate analyses. In addition, we calculated sensitivities and specificities with 95% CI for a fatal outcome, along with positive and negative likelihood ratios (LR+, LR-), respectively, in the presence or absence of each criterion. In the case of neonates referred twice, we used only the final referral for this calculation (which is synonymous with the total number of children ever referred). An LR+ is defined as (sensitivity)/(1-specificity); an LR- is defined as (1-sensitivity)/(specificity).^9^

## RESULTS

The intervention TBAs conducted a total of 2,007 deliveries, of which 38 were stillbirths (1.9%). Among live-born neonates, we had complete follow-up data on 1,889/1,969 (95.9%) (Figure). Table 1 summarizes baseline characteristics of the mothers and the 1,889 neonates who comprised the analysis set, stratified by the neonates' final vital status by day 28 of life (that is, alive vs. dead). Maternal baseline characteristics were similar between the 2 groups, except that more mothers of surviving neonates had been dewormed during pregnancy. Surviving neonates (n = 1,846) were significantly more likely to be female or to have been exclusively breastfed during the study period than those who died (n = 43). The gestational age of surviving neonates also trended lower, but the difference was not statistically significant.

**Figure.**
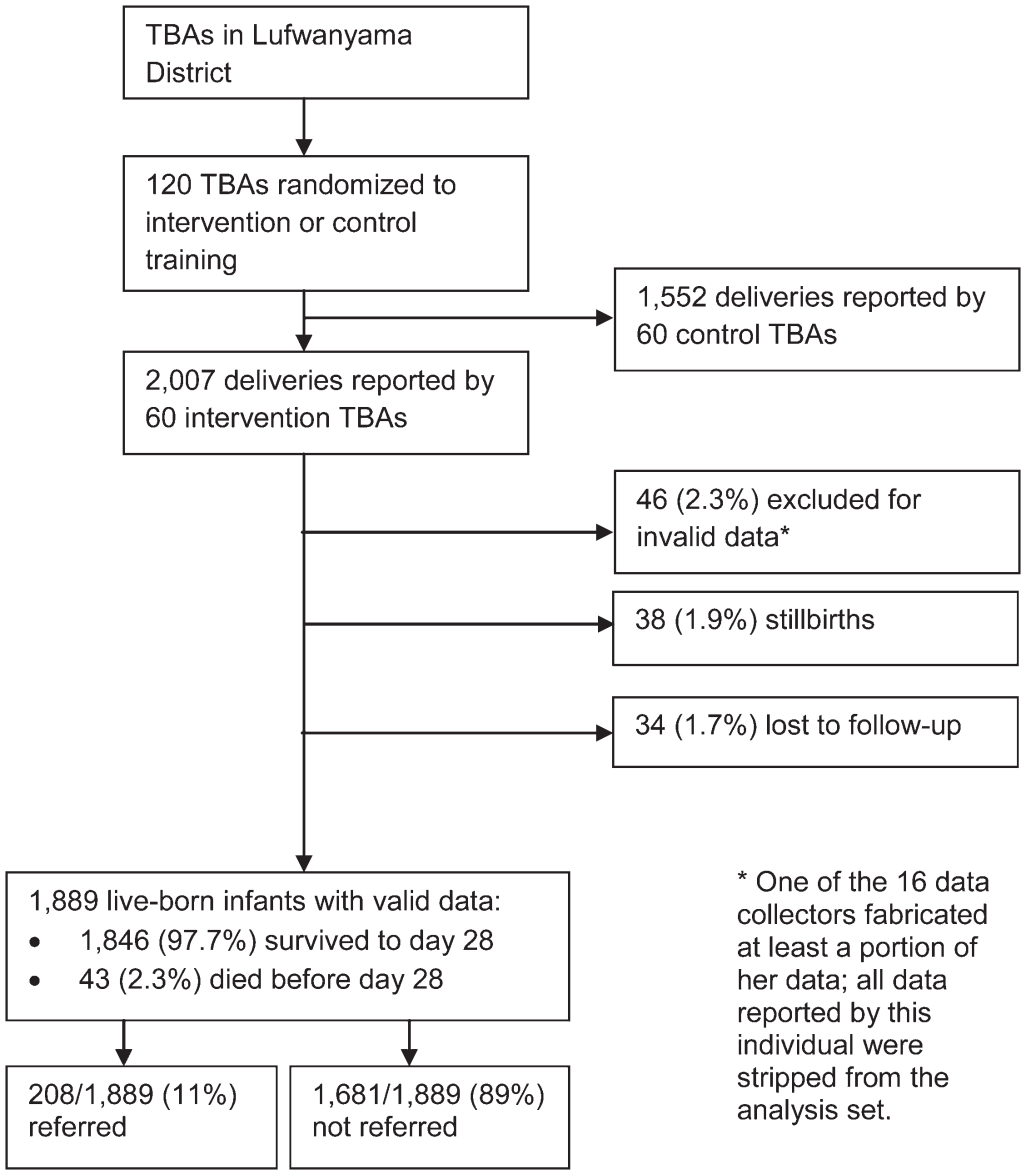
Data on Live-Born Neonates, June 2006–November 2008

**Table 1.** Baseline Maternal and Infant Characteristics Stratified by Infant Vital Status at Day 28 of Life

**Characteristic**	**Infant Survivors (n = 1,846)**	**Infant Deaths (n = 43)**	**All Neonates (N = 1,889)**	***P* Value**
**Maternal Characteristics**				
Age, y, mean (SD)	25.3 (0.15)	25.7 (1.27)	25.3 (0.15)	.77
Education (highest level attained), %				.41
None	16.7	20.9	16.8	
Some primary	69.2	58.1	68.9	
Some secondary	13.8	20.9	14.0	
Some higher	0.3	0.0	0.3	
Marital status, %				.84
Married	89.4	86.0	89.3	
Widowed	0.8	0.0	0.8	
Separated/divorced	2.5	4.7	2.5	
Never married	7.3	9.3	7.4	
No. of ANC visits attended, mean (SD)	3.3 (0.03)	3.2 (0.21)	3.3 (0.03)	.67
Receipt of treatment				
IPT of malaria with SP, %	89.8	83.7	89.6	.20
Deworming treatments, %	65.9	55.8	65.6	.01
Folic acid supplementation, %	85.5	95.3	85.8	.07
Iron supplementation, %	92.5	90.7	92.5	.65
Tetanus toxoid, %	72.8	65.1	72.6	.26
**Infant Characteristics**				
Female, %	50.5	32.6	50.1	.02
Gestational age at birth, weeks, mean (SD)	38.0 (0.31)	43.0 (2.19)	38.1 (0.31)	.33
Exclusively breastfed, %	96.8	86.0	96.6	<.001

Abbreviations: ANC, antenatal care; IPT, intermittent preventive therapy; SD, standard deviation; SP, sulfadoxine-pyrimethamine.

Table 2 summarizes characteristics of the TBAs. Most TBAs were married, identified farming as their primary occupation, and had worked as TBAs for an average of 6.3 years. Their educational backgrounds were quite limited: 83.3% either had no formal schooling or had failed to advance beyond primary school.

**Table 2.** Characteristics of the Intervention TBAs (N = 60)

**Characteristic**	****
Female, %	100
Age, y, mean (SD)	49.2 (0.79)
Years working as TBA, mean (SD)	6.3 (0.81)
Education	
Total years of education, mean (SD)	6.3 (0.48)
Never attended school, %	5.0
Primary education only, %	78.3
Main occupation, %	
TBA	1.7
Farmer	98.3
Source(s) of training prior to LUNESP,^a^ %	
Trained by family	11.5
Trained by community, not family	42.6
Trained by Lufwanyama DHMT	60.3
Trained by another government organization	33.3
Trained by an NGO	32.7

Abbreviations: DHMT, district health management team; LUNESP, Lufwanyama Neonatal Survival Project; NGO, nongovernmental organization; SD, standard deviation; TBA, traditional birth attendant.

a TBAs often received training from more than 1 source.

Of the 1,889 neonates, 208 (11.0%) were referred to an RHC, and 11 (0.6%) were referred twice. Thus, a total of 219 referrals occurred, 113 during week 1 (51.6% of referrals) and 106 during weeks 2–4 (48.4% of referrals). The majority of neonates (176/208 [84.6%]) completed their referral to the RHC. TBAs administered amoxicillin 188 times, 99 times during week 1 (52.7% of total administrations) and 89 times (47.3% of total administrations) during weeks 2–4. Among referred neonates, 171/208 (82.2%) received amoxicillin; among neonates given amoxicillin, 171/183 (93.4%) were referred (referral status unknown for 5 subjects).

Referred neonates had been ill for a median of 2 days prior to the TBAs' evaluation. TBAs cited a median of 3 criteria to justify their referrals, with a range of 0 (8 times) to 8 (2 times) reasons. A single reason for referral was cited 19 times; 2 reasons, 23 times; 3 reasons, 52 times; 4 reasons, 73 times; and 5 or more reasons, 41 times. The 6 most common reasons for referral, alone or in combination, were because: (1) the TBA thought the neonate “appeared ill” (82.8%), (2) the mother thought the neonate “appeared ill” (80.8%), (3) “felt hot” (41.2%), (4) “had a cough” (40.4%), (5) “was not making urine” (31.9%), or (6) was having “difficulty breathing” (26.4%).

Table 3 summarizes the accuracy for the 16 referral criteria for identifying neonates who subsequently died. Individually, none displayed both high sensitivity and high specificity. Moreover, several signs were nearly ubiquitous in their presence and some were tautological. For example, 95% of referred neonates included the criterion that the TBA thought the baby “appeared ill,” but logically a TBA would be unlikely to refer otherwise. Moreover, this criterion was never cited alone but was always accompanied by other, more specific criteria. Criteria that were cited frequently were “felt hot/cold,” “had cough,” “refused to feed,” had “difficulty breathing,” and “not making urine.” Conversely, “rapid breathing,” “bulging fontanel,” and “infected umbilicus” were rarely cited, and no neonates were referred for “diarrhea” or “chest wall in-drawing.”

**Table 3.** Sensitivity, Specificity, and Likelihood Ratios of Specified Reasons for Referral at Predicting a Fatal Outcome for the Referred Infant

**Reason for Referral^a^**	**Times Cited**	**Prevalence % (95% CI)**	**Sensitivity % (95% CI)**	**Specificity % (95% CI)**	**LR+**	**LR-**
Fever or felt hot	82	41.2 (34.3–48.4)	15.0 (3.2–37.9)	55.9 (48.3–63.3)	0.3	1.5
Had cough	80	40.4 (33.5–47.6)	25.0 (8.7–49.1)	57.9 (50.3–65.2)	0.6	1.3
Diarrhea	15	7.6 (4.3–12.2)	0.0 (0.0–16.1)	91.6 (86.5–95.2)	0.0	1.1
Refusing to feed	24	12.1 (7.9–17.4)	45.0 (23.1–68.5)	91.6 (86.6–95.2)	**5.4**	0.6
Sleepy or difficult to arouse	8	4.0 (1.8–7.8)	20.0 (5.7–43.7)	97.8 (94.3–99.4)	**9.1**	0.8
Floppy or poor muscle tone	18	9.1 (5.5–14.0)	25.0 (8.7–49.1)	92.7 (87.8–96.1)	**3.4**	0.8
Not making urine	22	31.9 (21.2–44.2)	57.1 (18.4–90.1)	71.0 (58.1–81.8)	2.0	0.6
Convulsions, fits, or seizures	8	4.1 (1.8–7.8)	15.0 (3.2–37.9)	97.2 (93.5–99.1)	**5.4**	0.9
Difficulty breathing	52	26.4 (20.4–33.1)	65.0 (40.8–84.6)	78 (71.1–83.8)	**3.0**	**0.4**
Rapid breathing	5	2.5 (0.8–5.8)	10.0 (1.2–31.7)	98.3 (95.1–99.6)	**5.9**	0.9
Chest wall in-drawing	1	0.5 (0.0–2.8)	0.0 (0.0–16.1)	99.4 (96.9–100.0)	0.0	1.0
Skin pustules or red rash	6	3.0 (1.1–6.5)	0.0 (0.0–39.0)	96.6 (92.8–98.7)	0.0	1.0
Infected umbilicus	3	1.5 (0.3–4.4)	5.0 (0.1–24.9)	98.9 (96–99.9)	**4.5**	1.0
Bulging fontanel	5	2.5 (0.8–5.8)	10.0 (1.2–31.7)	98.3 (95.1–99.6)	**5.9**	0.9
TBA thought baby appeared ill	164	82.8 (76.8–87.8)	95.0 (75.1–99.9)	18.5 (13.1–25.0)	1.2	**0.3**
Mother thought baby appeared ill	160	80.8 (74.6–86.0)	80.0 (56.3–94.3)	19.1 (13.6–25.7)	1.0	1.0
Other^b^	62	NA	NA	NA	NA	NA
No reason cited	8	NA	NA	NA	NA	NA

Abbreviations: CI, confidence interval; LR+ and LR−, positive and negative likelihood ratios (clinically relevant LR+ and LR− values are in bold); NA, not applicable.

a TBAs were free to specify more than 1 reason for a given referral, so the total number of reasons for referral exceeds the number of neonates who were referred (208).

b Among the “other” reasons cited, those cited more than once included 12 citations for abdominal complaints (not making stool, swollen or tender belly, or diarrhea); 8 citations for inconsolable crying; 6 because the baby had been resuscitated at birth (all of which occurred during the first follow-up visit during week 1); 6 for skin rashes or sores; 4 for congenital defects or prematurity; 3 for respiratory complaints; and 3 for eye infections or discharge.

Overall, the most useful sign for both predicting and excluding a fatal outcome was the criterion “difficulty breathing,” with sensitivity of 65% and specificity of 78%, yielding positive and negative LRs of 3.0 and 0.4, respectively. Infrequent signs that predicted a fatal outcome were: “baby sleepy/unarousable” (LR+ 9.1), “rapid breathing” (LR+ 5.9), “bulging fontanel” (LR+ 5.9), “refuses to feed” (LR+ 5.4), “convulsions/fits/seizures” (LR+ 5.4), “infected umbilicus” (LR+ 4.5), and “floppy/poor muscle tone” (LR+ 3.4). Conversely, the only criterion strongly predictive of infant survival was if the TBA did not feel that the infant looked sick, where the LR- was 0.3. The opinion of the mother was neither sensitive nor specific (LR+/− both 1.0).

Among live-born neonates, 43 (2.3%) died during the first 28 days of life. Among referred neonates, 9.6% (20/208) ultimately died. By contrast, among neonates who were not referred, only 1.2% died (22/1,658), with 1 neonate whose referral status was missing. Since a neonate who dies shortly after birth has little opportunity to be referred, we assessed referral status relative to the timing of deaths. Of the non-referred deaths, 10/22 (45%) occurred on the first day of life, while only 4/20 (20%) of the referred deaths occurred on the first day of life.

Neonates sick enough to warrant referral (regardless of whether given amoxicillin or not) were nearly 8 times more likely to die than babies who were not referred (RR = 7.93, 95% CI = 4.4–14.3). Similarly, neonates deemed sick enough to receive amoxicillin (regardless of referral status) were nearly 5 times likely to die as those not given amoxicillin (RR = 4.7, 95% CI = 2.5–8.7).

Neonates sick enough to warrant referral were nearly 8 times more likely to die than babies who were not referred.

The TBAs recorded a subjective impression of illness severity for 185/208 referred neonates (88.9%), as summarized in Table 4. Neonates judged as “extremely sick” by the referring TBA were far more likely to die than those deemed “not sick” or only “moderately sick” (RR = 8.61, 95% CI = 4.0–18.5).

**Table 4.** Survival of Neonates Stratified by the Subjective Severity of Illness Rating Assigned by the Referring TBA (N = 185)^a^

**Outcome**	**Severity Rating, n/N (%)**		
	**Not sick**	**Moderately sick**	**Extremely sick**
Died	3/49 (6.1%)	6/113 (5.3%)	11/23 (47.8%)
Survived	46/49 (93.9%)	107/113 (94.7%)	12/23 (52.2%)
Total	49/185 (26.5%)	113/185 (61.1%)	23/185 (12.4%)^b^

Abbreviation: TBA, traditional birth attendant.

a A total of 208 neonates were referred; TBAs provided a severity assessment for 185 of the 208 neonates (88.9%). Data for analysis relates only to the final referral if the infant was referred more than once given that an infant referred twice could not possibly have died during the first referral event.

b Chi square = 37.3 with 2 df, *P*< .001; comparing “extremely sick” vs. combined (“not sick” and “moderately sick”), RR of fatal outcome = 8.61, 95% CI = 4.0–18.5.

Neonates deemed “extremely sick” by the referring TBA were about 9 times more likely to die than neonates judged less severely ill.

## DISCUSSION

In LUNESP, about 11% of neonates cared for by the intervention TBAs were targeted for AFR, a rate that is consistent with the incidence of serious bacterial infections during the first 28 days of life.^10^ Notably, neonates who were referred were approximately 8 times more likely to have a fatal outcome than those who did not, and those who received amoxicillin were nearly 5 times more likely to die. It makes little sense that referral in itself causes death and implausible that a safe and well-tolerated antibiotic like amoxicillin would increase mortality. Therefore, a more logical interpretation is that referral and/or use of amoxicillin were both markers of very ill children at high risk of death. This interpretation is supported by the observation that neonates deemed “extremely sick” by the referring TBA were about 9 times more likely to die than neonates judged less severely ill. Taken together, we conclude that the TBAs demonstrated sound clinical judgment and reacted according to their training, which strongly supports the feasibility of using TBAs in this role.

With that said, in order for an intervention like AFR to be effective, several conditions must be met. First, the CHW must have sufficient clinical judgment to accurately identify those children who are in crisis. Second, they must be empowered, trained, and equipped to act upon that determination. Third, the actions that ensue as a consequence must be sufficient to interrupt the disease process that would ultimately lead to death.

Our analysis suggests that the first 2 conditions were met, but evidence for the third is more ambiguous. In our main effects analysis, during weeks 2–4 of life, the period during which AFR would presumably have been most effective (and the effects of NRP minimal), mortality among neonates cared for by intervention TBAs was reduced by about 50%, but this did not reach statistical significance.^6^ Our study had been powered to detect an overall effect on mortality due to the combination of NRP and AFR, so the infrequency of sepsis could have left us underpowered to isolate the effect of AFR. This possibility could be evaluated in a larger clinical trial. However, another possibility is that the receiving RHCs were insufficiently prepared to manage neonatal sepsis, which would be an essential pre-condition for the third condition listed above. If so, 2 potential solutions are suggested.

Rural health clinics may not be sufficiently prepared to manage neonatal sepsis.

First, one could focus resources on strengthening the “back end” of the referral process, that is, the receiving RHCs. In this approach, one would continue to limit the role of TBAs to giving a first dose of antibiotics and facilitating referral, essentially using the TBAs to extend the reach of the RHCs into the community while focusing resources to strengthen the capacity of the RHCs to manage newborns with serious bacterial infections.

Alternatively, one could invest in strengthening the “front-end” by increasing the capacity of the TBAs themselves, following the model of Bang and Bang in India.^11^^–^^13^ There, village health workers were responsible not just for identifying sepsis but also for administering the full antibiotic treatment course in the community using a combination of oral and injectable antibiotics. This approach is ambitious since it requires that CHWs be trained to use injectable antibiotics dosed according to infant body weight and that systems be established for managing contaminated sharps, storing antibiotics appropriately, and for reclaiming expired drugs. However, this strategy minimizes the delay between identification of a sick child and the start of definitive antibiotic therapy, and it largely eliminates the problem of non-adherence to referrals.

One distinction: the CHWs from studies in South Asia had significantly more training than the LUNESP TBAs, both in terms of their background education and in the intervention training specifically.^14^^–^^16^ Even so, a hybrid of these 2 models might yet be feasible: TBAs could be responsible for identifying neonates with possible sepsis, initiating therapy, and referring, but the single-dose amoxicillin could be replaced with broader spectrum antibiotics with longer half-lives, or combinations of drugs such as the oral cotrimoxazole and injectable gentamicin used by Bang and Khanal.^12^^,^^16^ Decisions about which strategy is most appropriate will depend on the capacity of the CHWs, the local epidemiology of neonatal sepsis, the capacity of RHCs, and especially the average transit times to the RHCs. Logically, the strategy of embedding more capacity in TBAs or other CHWs becomes more attractive as distances to the RHCs increase.

Solutions might combine boosting capacity of RHCs with expanding TBAs' treatment capabilities in the field.

Several other findings in this analysis were of interest. First, one of the most common reasons cited for initiating AFR was that the TBA thought the neonate “appeared ill.” This criterion had been included to empower the TBA to initiate AFR based on general clinical impressions, absent more specific definable signs or symptoms.^8^ Notably, among referred neonates who did not meet this criterion, mortality was about one-third lower. This lends further credibility to the clinical judgment of the TBAs and is consistent with other researchers' experience with more highly skilled CHWs in Bangladesh, India, and Nepal.^14^^–^^16^ By contrast, the criterion “mother thought infant looked sick” was neither sensitive nor specific.

One of the most common reasons why TBAs initiated AFR was because they thought the neonate “appeared ill.”

Second, the most clinically useful criterion was “difficulty breathing.” This criterion predicted mortality when present (LR+ 3.0), and it was protective when absent (LR- 0.4). Conversely, “rapid breathing” (a sign of “severe pneumonia”) was rarely cited, and “chest wall in-drawing” (a criterion for “very severe pneumonia”), was never cited. Both signs require some skill to identify, and, in the case of rapid breathing, a timer to count respiratory rates, so their absence as reasons for referral by TBAs should be interpreted cautiously. The clinical sign that most strongly predicted a fatal outcome was “difficulty arousing the child,” with a 10-fold increased likelihood of death. It is relevant that several of the referral criteria that performed best in the hands of TBAs were included in the young infant integrated management of childhood illness algorithm and validated in the Young Infant Clinical Signs Study.^17^^,^^18^

Third, the AFR training appeared successful at coupling the actions of referring and administering amoxicillin. In more than 80% of instances, the one behavior predicted the other. While one can lament the fact that roughly one-fifth of referrals were not completed, this rate is comparatively high relative to other similar community intervention strategies of this kind.^19^^,^^20^ Nonetheless, it is clearly a vulnerable point in the AFR strategy. While our data do not indicate the reason for failure to complete a referral, previous research has suggested that mothers may decline referral for a variety of reasons, including distance to facility, financial barriers, and reluctance to comply in the absence of a spouse's authorization.^15^^,^^19^^,^^21^^–^^24^

A general limitation of this analysis was that despite having very complete data from the TBAs about the reasons for referral, data were not collected from the RHCs, which were outside of the LUNESP study. Future studies should assess both sides of the referral system, and also assess the average elapsed time between a TBA's first contact with a sick infant and the first dose of amoxicillin, as well as average transit times to the nearest RHC.

Despite these limitations, our findings provide cause for optimism. Can TBAs be trained to identify sick neonates, refer them, and initiate treatment at the community level? The answer is “yes.” Whether this translates into reduced mortality is a testable question. Moreover, these results should be highly generalizable. The LUNESP TBAs were relatively advanced in that they had all received formal training in obstetrical care prior to this study, but they were not highly educated (83% never advanced beyond primary school), and most were illiterate, requiring that our trainings be done without text materials. As such, our study demonstrates that lack of formal education does not mean that individuals are unintelligent or un-trainable. Quite the opposite: despite their lack of formal schooling, the Lufwanyama TBAs proved to be eager and successful students, capable of mastering complex concepts and acting upon them appropriately. Our experiences justify further investigations to assess the effectiveness of community-based identification and presumptive treatment of sepsis in the hands of a relevant cadre of CHWs who, by dint of their obstetrical work, have close access to newborns during their period of greatest vulnerability.

TBAs can be trained to identify sick neonates, initiate treatment, and refer them.

## Notes

**Peer Reviewed**
